# Robust ellipsoidal set-membership fault estimation for time-varying systems with uniform quantization effects over sensor networks

**DOI:** 10.7717/peerj-cs.872

**Published:** 2022-03-17

**Authors:** Peiying Zhao, Jianxi Zhang

**Affiliations:** 1 School of Mathematics and Quantitative Economics, Shandong University of Finance and Economics, Jinan, Shandong, China; 2 Shandong Institute of Commerce and Technology, Jinan, China; 3 National Engineering Research Center for Agricultural Products Logistics, Jinan, China

**Keywords:** Set-membership fault estimation, Time-varying systems, Uniform quantization, Unknown-but-bounded noises, Sensor networks

## Abstract

This article is concerned with the robust set-membership fault estimation problem for a class of uncertain discrete time-varying systems over sensor networks. The system measurements are subject to the uniform quantization which results in the unknown-but-bounded noises. Attention is focused on the design of a set-membership fault estimator such that, in the simultaneous presence of uncertain parameters, unknown-but-bounded noises and uniform quantization effects, the estimation errors are confifined to a certain ellipsoidal region. By using the mathematical induction, a suffificient condition is derived for the existence of the desired fault estimator at each time step in terms of a set of recursive matrix inequalities. Moreover, an optimization problem is formulated by minimizing the ellipsoid of the estimation error. Finally, two numerical examples are provided to illustrate the effectiveness of the proposed fault estimator design scheme.

## Introduction

A sensor network consists of a large number of sensor nodes with limited sensing, communication and computation capabilities. These sensor nodes are usually distributed geographically in certain areas and coordinated to execute tasks by exchanging measurements with neighboring sensors according to a given topology. During the past decades, increasing research attention has been devoted to sensor networks due primarily to the fact that they have been extensively applied in many fields such as military sensing, agricultural production, environmental monitoring and intelligent buildings (Chen et al., 2014; Kampianakis et al., 2014; Maurya & Jain, 2016). In order to estimate the states of plants *via* distributed sensors, the problem of distributed filtering for sensor networks has gained considerable research interest and many filter design algorithms have been reported in the literature, see *e.g.*, (Niu et al., 2021; Zhang et al., 2014; Zou et al., 2019; Sheng, Niu & Gao, 2019). Due to unexpected changes and bad working environment, there may appear various faults during the application of sensor networks. Unfortunately, owing to the mathematical complexities, the corresponding results on fault estimation problem over sensor networks have been very few in comparison with the fruitful results on the distributed filtering problem. With the growing demand for reliability and safety in modern control systems, the fault diagnosis problem has attracted much research attention in recent years (Ding, 2013; Gao et al., 2019; Mhdawi & Al-Raweshidy, 2020; Zhong et al., 2020; Zhou et al., 2020). It is well known that, almost all real-time systems contain some time-varying parameters. For time-varying systems, the fault estimation can provide the shape and size of the faults *via* available measurements, and it is important for the active fault tolerant control and has thus stirred remarkable interest (Sheng et al., 2017b; Dong et al., 2016; Gao, Yang & Sheng, 2019; Ren, Wang & Lu, 2017; Zhong et al., 2010; Gao et al., 2020; Zhang, Jiang & Shi, 2017). For example, the finite-horizon *H*_*∞*_ fault estimation problem has been studied in Dong et al. (2016) for nonlinear time-varying systems with fading channels and randomly occurring faults. The parity space-based fault estimation problem has been investigated in Zhong et al. (2010) for linear discrete time-varying systems, and the design of fault estimator has been formulated as to find the minimum for a matrix quadratic form. However, the problem of fault estimation has not been adequately addressed so far for time-varying sensor networks, which gives rise to the first motivation of this paper.

In real-world sensor networks, the information received by sensors is often quantized before being transmitted to filters because filters are usually digital devices and can only process digital signals. Generally, the quantized communication models are mainly categorized as logarithmic quantization (Fu & Xie, 2005; Han, Liu & Yang, 2016; Li, Lin & Zhang, 2017) and uniform quantization (Li, Ho & Lu, 2013; Rojas & Lotero, 2015; Sheng et al., 2017a; Zou et al., 2020). The logarithmic quantization model partitions the state space in a logarithmic way, and the quantization level becomes smaller in regions which are closer to the origin. In Fu & Xie (2005), the logarithmic quantization effect has been converted into the norm-bounded uncertainty which can be effectively handled by the robust control theory. The uniform quantization model provides a uniform partition of the state space, and the lengths of each two quantization regions are equal. The uniform quantization error is usually modeled as the unknown-but-bounded noise which is neither Gaussian nor energy-bounded, then it cannot be dealt with by traditional Kalman filtering or *H*_*∞*_ filtering methods. This may explain why reports on problems of filter design under uniform quantization effects have been scattered compared with those under logarithmic ones. In Rojas & Lotero (2015), the problem of deriving the infimal signal-to-noise-ratio has been investigated for stabilizability subject to channel input quantization, where the uniform quantization error process is assumed to obey uniform distribution. In Sheng et al. (2017a), the input-to-state stability in probability has been analyzed for a general class of nonlinear stochastic systems with uniform quantization effects. On the other hand, the modeling errors are often encountered in reality and the designed fault estimation schemes should be robust against parameter uncertainties. Up to now, the fault estimation problem for sensor networks with uniform quantization effects has not been studied yet, not to mention the case when the parameters subject to uncertainties.

In order to investigate the filter problem of systems affected by unknown-but-bounded noises, the set-membership filtering method, which introduced a support function to characterize the set of possible states consistent with given measurements, has been proposed in Witsenhausen (1968), and such a method has been widely employed later on Ghaoui & Calafiore (2001), Wei et al. (2015), Yang & Li (2009). In Ghaoui & Calafiore (2001), the convex optimization method has been proposed to handle the set-membership filtering for discrete-time systems with bounded noise and parameter uncertainty. In Yang & Li (2009), the recursive scheme has been derived for the design of set-membership filters with sensor saturation by solving the time-varying linear matrix inequality. Recently, some fault diagnosis methods have been developed following the set-membership approach (Chatti et al., 2016; Reppa & Tzes, 2016; Tornil-Sin et al., 2012). For instance, a fault diagnosis strategy has been proposed in Reppa & Tzes (2016) for capturing multiple abrupt parametric faults in a system based on set-membership identification. In Chatti et al. (2016), the fault diagnosis problem has been presented as a constraint satisfaction problem which can be solved by the set-membership approach. It is worth mentioning that, despite the recent progress in the set-membership filter/fault diagnosis, it remains an open problem to develop the set-membership fault estimation mechanism for large-scale distributed systems such as sensor networks, and this constitutes another one of the motivations for our current study.

Summarizing the above discussions, in this paper, we aim to study the problem of robust set-membership fault estimation for time-varying sensor networks with uniform quantization effects. The novelties of this paper are highlighted as follows. (1) The set-membership fault estimation problem is investigated for time-varying systems over sensor networks; (2) The system model addressed is comprehensive to cover uncertain parameters, unknown-but-bounded noises as well as uniform quantization effects, hence reflecting the reality more closely. (3) The developed fault estimator design algorithm is suitable for online applications *via* solving a set of recursive linear matrix inequalities (RLMIs).

The rest of the paper is organized as follows. In “Problem Formulation and Preliminaries”, the time-varying system over sensor networks with uniform quantization effects is introduced, and the problem under consideration is formulated. In “Main Results”, the set-membership fault estimator design problem is solved in terms of the solution to a set of RLMIs. Moreover, two simulation example are provided in “Numerical Example” to show the effectiveness of the main results derived. Finally, conclusions are drawn in “Conclusion”.

**Notations.** The notation used here is fairly standard except where otherwise stated. ${\rm {\mathbb R}}$ (respectively, ${\rm {\mathbb N}}$) is the set of all real numbers (respectively, non-negative integers). ${{\rm {\mathbb R}}^n}$ is the set of all real *n*-dimensional vectors, and ${{\rm {\mathbb R}}^{m \times n}}$ is the set of all *m* × *n* real matrices. The notation *X* ≥ *Y* (*X* > *Y*), where *X* and *Y* are real symmetric matrices, means that *X* − *Y* is positive semi-definite (respectively, positive definite). *A*^*T*^ denotes the transpose of a matrix *A*. ${\parallel {x}\parallel}$ describes the Euclidean norm of a vector *x*. tr{·} stands for the trace of a matrix. For matrices $A \in {{\rm {\mathbb R}}^{m \times n}}$ and $B \in {{\rm {\mathbb R}}^{p \times q}},$ their Kronecker product is a matrix in ${{\rm {\mathbb R}}^{mp \times nq}}$ represented by ${A\otimes B}$. $I_n$ and 0_*n*_ denote identity matrix and zero matrix of *n* dimensions, respectively. $1_n$ denotes an *n*-dimensional column vector with all ones. $\rm diag{\{}{\cdots}{\}}$ is a block-diagonal matrix.

## Problem Formulation and Preliminaries

The sensor network has *N* sensor nodes which are distributed in the space according to a specific interconnection topology characterized by a directed graph ${\rm {\mathcal{O}}} = \{ {\rm {\mathcal{V}}},{\rm {\mathcal{E}}},{\rm {\mathcal{A}}}\}$ with the set of nodes ${\rm {\mathcal{V}}} = \{ 1,2, \cdots ,N\}$, the set of edges ${\rm {\mathcal{E}}} \subseteq {\rm {\mathcal{V}}} \times {\rm {\mathcal{V}}},$ and the weighted adjacency matrix ${\rm {\mathcal{A}}} = [{a_{ij}}] \in {{\rm {\mathbb R}}^{N \times N}}$ with nonnegative elements *a*_*ij*_. An edge of ${\rm {\mathcal{O}}}$ is represented by an ordered pair (*i*, *j*), and ${a_{ij}} \gt 0 \Leftrightarrow (i,j) \in {\rm {\mathcal{E}}}$ which means that sensor *i* can receive information from sensor *j*. Assume that *a*_*ii*_ = 1, $i \in {\rm {\mathcal{V}}},$ and then, (*i*, *i*) is regarded as an additional edge. The set of neighbors of node $i \in {\rm {\mathcal{V}}}$ plus the node itself is denoted by ${{\rm {\mathcal{N}}}_i} = \{ j \in {\rm {\mathcal{V}}}|(i,j) \in {\rm {\mathcal{E}}}\} .$

Consider the following time-varying system

(1) $$x(k + 1) = (A(k) + \Delta A(k))x(k) + B(k)f(k) + C(k)v(k)$$

with *N* sensor nodes modeled by

(2) $${y_i}(k) = {\kern 1pt} q({D_i}(k)x(k)) + {E_i}(k)w(k),\quad i = 1,2, \ldots ,N,\;k \in {\rm {\mathbb N}}$$

where $x(k) \in {{\rm {\mathbb R}}^n}$ is the system state, $f(k) \in {{\rm {\mathbb R}}^f}$ is the fault signal, and ${y_i}(k) \in {{\rm {\mathbb R}}^p}$ is the output measured by sensor *i*. *A*(*k*), *B*(*k*), *C*(*k*), *D*_*i*_(*k*) and *E*_*i*_(*k*) are known, real, time-varying matrices with appropriate dimensions. $\Delta A(k)$ denotes the time-varying norm-bounded parameter uncertainty which satisfies ${\Delta A(k)} = {M_A(k) L_A(k) N_A(k)}$ where *M*_*A*_(*k*) and *N*_*A*_(*k*) are known time-varying matrices of appropriate dimensions, and *L*_*A*_(*k*) is an unknown matrix function satisfying $L_A^T(k){L_A}(k) \le I,$ $\forall k \in {\rm {\mathbb N}}.$ $v(k) \in {{\rm {\mathbb R}}^{{n_v}}}$ and $w(k) \in {{\rm {\mathbb R}}^{{n_w}}}$ denotes the process and measurement noises, respectively, which are assumed to be unknown-but-bounded.

In a sensor network, the measurement output *y*_*i*_(*k*) of sensor *i* is usually quantized when it is transmitted to the neighbors, and the uniform quantizer *q*(·) is defined as

$$q( \cdot ) = \left[ {{q_1}\left( {{u^{(1)}}} \right),\;\;{q_2}\left( {{u^{(2)}}} \right),\;\; \cdots ,\;\;{q_m}\left( {{u^{(m)}}} \right)} \right]$$

where *u*^(*j*)^ denotes the *j*th entry of the vector $u \in {{\rm {\mathbb R}}^m}$ and

(3) $${q_j}\left( {{u^{(j)}}} \right) \triangleq \varepsilon \left[ {\displaystyle{{{u^{(j)}}} \over \varepsilon }} \right]$$

with [·] being the function that rounds a real number to the nearest integer and *ε* being called a quantizing level. The quantization range is supposed to be sufficiently large and the quantization error is defined by ${\delta ^{(j)}} \triangleq {q_j}\left( {{u^{(j)}}} \right) - {u^{(j)}}$ and it is bounded by *ε*/2, *i.e.*,

(4) $$\left| {{\delta ^{(j)}}} \right| \le \varepsilon /2.$$

**Assumption 2.1** *The noise sequences v(k) and w(k) are confined to the following ellipsoidal sets:*

(5) $$\left\{ {\matrix{ {} \hfill  {{\rm {\mathcal{V}}}(k) \triangleq \{ v(k):{v^T}(k){S^{ - 1}}(k)v(k) \le 1\} } \hfill \cr {} \hfill  {{\rm {\mathcal{W}}}(k) \triangleq \{ w(k):{w^T}(k){T^{ - 1}}(k)w(k) \le 1\} } \hfill \cr } } \right.$$

where *S*(*k*) and *R*(*k*) are known positive definite matrices with compatible dimensions.

**Assumption 2.2** *The dynamic characteristics of the fault f(k) can be described by*

(6) $$f(k + 1) = (F(k) + \Delta F(k))f(k)$$

where *F*(*k*) is a known time-varying matrix and $\Delta F(k) = {M_F(k)L_F(k)N_F(k)}$ represents the parameter uncertainty with *M*_*F*_(*k*), *N*_*F*_(*k*) being known time-varying matrices and *L*_*F*_(*k*) satisfying *L*^*T*^_*F*_(*k*)*L*_*F*_(*k*) ≤ *I*.

Letting $\bar x(k) = [{x^T}(k)\;\;{f^T}(k{)]^T} \in {{\rm {\mathbb R}}^{{n_f}}}$ with *n*_*f*_ = *n* + *f*, it follows from (1), (2) and (6) that

(7) $$\left\{ {\matrix{ {}{\bar x(k + 1) = \left( {\bar A(k) + \Delta\bar A(k)} \right)\bar x(k) + \bar C(k)v(k)} \cr {\hskip1pc}{{y_i}(k) = q({{\bar D}_i}(k)\bar x(k)) + {E_i}(k)w(k),\quad i = 1,2, \ldots ,N} \cr } } \right.$$

where

$$$\bar A(k) = \left[ {\matrix{ {A(k)} & {B(k)} \cr 0 & {F(k)} \cr } } \right],\;\Delta\bar A(k) = \left[ {\matrix{ {\Delta A(k)} & 0 \cr 0 & {\Delta F(k)} \cr } } \right],\;\bar C(k) = \left[ {\matrix{ {C(k)} \cr 0 \cr } } \right],\;{\bar D_i}(k) = [{D_i}(k)\;\;0].$$$

Moreover, we have

(8) $$\Delta\bar A(k) = \bar M(k)\bar L(k)\bar N(k),$$

where $\bar M (k) = {\rm diag}{\{}{M_A(k), M_F(k)}{\}},\; \bar L (k) = {\rm diag}\{L_A(k), L_F(k)\},$ $\bar N (k) =   {\rm diag}\{N_A(k), N_F(k)\} \;{\rm and}\; \bar L (k) \;{\rm satisfies}\; \bar L^T(k) \bar L (k) \leq \ I.$

Consider the following estimator on the *i*th sensor node as follows:

(9) $${\hat {\bar x}_i}(k + 1) = {\kern 1pt} \bar A(k){\hat {\bar x}_i}(k) + \sum\limits_{j \in {{\rm {\mathcal{N}}}_i}} {a_{ij}}{G_{ij}}(k)({y_j}(k) - {\bar D_j}(k) {\hat{\bar x}_j}(k)),\quad {\hat {\bar x}_i}(0) = 0$$

where ${\hat {\bar x}_i}(k) = [{\hat x_i}(k)\;\;\hat f(k{)]^T} \in {{\rm {\mathbb R}}^{{n_f}}}$ with ${\hat x_i}(k)$ and $\hat f(k)$ being the estimates of the state *x*(*k*) from sensor node *i* and the fault *f*(*k*), respectively. ${G_{ij}}(k) \in {{\rm {\mathbb R}}^{{n_f} \times p}}$ are the estimation parameters to be determined.

**Assumption 2.3** *The initial state $\bar{x}(0)$ satisfies*

(10) $${\bar x^T}(0){P^{ - 1}}(0)\bar x(0) \le 1$$

*where* $P(0) \in {{\rm {\mathbb R}}^{{n_f} \times {n_f}}}$ *is a given positive definite matrix*.

By denoting ${\tilde x_i}(k) \triangleq \bar x(k) - {\hat {\bar x}_i}(k)$ and $\delta_i(k) = q\bar{D}_i(k) \bar{x} (k)) - \bar{D}_i(k) \bar{x} (k)$, we have the following estimation error system

(11) $$\eqalign{ {\tilde x_i}(k + 1) = {\kern 1pt} \left( {\bar A(k) + \Delta\bar A(k)} \right)\bar x(k) + \bar C(k)v(k) \cr- \Big(\bar A(k){\hat {\bar x}_i}(k) + \sum\limits_{j \in {{\rm {\mathcal{N}}}_i}} {a_{ij}}{G_{ij}}(k)({y_j}(k) - {\bar D_j}(k){\hat {\bar x}_j}(k))\Big) \cr= {\kern 1pt} \bar A(k){\tilde x_i}(k) + \Delta\bar A(k)\bar x(k) + \bar C(k)v(k) \cr- \sum\limits_{j \in {{\rm {\mathcal{N}}}_i}} {a_{ij}}{G_{ij}}(k) \Big({\bar D_j}(k){\tilde x_j}(k) + {\delta _j}(k) + {E_j}(k)w(k)\Big)}$$

where ${\delta _j}(k) \in {{\rm {\mathbb R}}^p}$ with

(12) $${\rm \parallel }{\delta _j}(k){{\rm \parallel }^2} \le \bar \varepsilon ,\quad \bar \varepsilon = \displaystyle{{{\varepsilon ^2}p} \over 4}.$$

**Remark 1** *In practice, the uniform quantization errors can be modeled as unknown-but-bounded noises which might be neither Gaussian nor energy-bounded. As such, the traditional filtering approaches, such as Kalman filtering and H*_*∞*_ *filtering, are no longer applicable. In search of an alternative methodology, the set-membership filtering appears to be an appropriate candidate for handling the unknown-but-bounded noises resulting from the uniform quantization error*.

**Remark 2** *The fault model* (6) *may represent a general class of faults. For example, the fault becomes a constant one when F(k) ≡ I and δ F(k) ≡ 0. In* Dong et al. (2016) *and* Ren, Wang & Lu (2017), *the finite-horizon H*_*∞*_ *fault estimation problem has been studied for time-varying systems with the fault model* (6). *Due to the existence of unknown-but-bounded noises v(k), w(k), and the uniform quantization error, the method proposed in* Dong et al. (2016) *and* Ren, Wang & Lu (2017) *cannot be used to solve the fault estimation problem for system* (1) *and* (2). *In this paper, the set-membership fault estimation problem will be investigated for time-varying systems with uniform quantization effects over sensor networks*.

The purpose of the problem addressed in this paper is stated as follows.

**Problem:** Considering system (1) and (2) and estimator (9), for a given sequence of constraint matrices *P*(*k*), design the sequence of estimator parameters *G*_*ij*_(*k*) such that the following requirement is met

(13) $${\Psi _i}(k) \triangleq \;\tilde x_i^T(k){P^{ - 1}}(k){\tilde x_i}(k) \le 1,\quad i = 1,2, \ldots ,N,\quad k \in {\rm {\mathbb N}}.$$

## Main Results

In this section, we will design an estimator of form (9) for system (1) and (2) subject to uncertain parameters, unknown-but-bounded noises and uniform quantization effects. First of all, we introduce the following lemmas which are needed in this paper.

**Lemma 1** *(S-procedure)* (Boyd et al., 1994) *Let* ${\phi_0(\cdot)} , {\phi_1}(\cdot),{\cdots}, {\phi_P}(\cdot)$ *be quadratic functions of the variable* $\varsigma \in {{\rm {\mathbb R}}^n}:\;{\phi _i} \triangleq {\varsigma ^T}{T_i}\varsigma \;(i = 0,1, \ldots ,p)$ *with* $T_i^T = {T_i}$. *If there exist* $\alpha1, \alpha2,{\cdots}, \alpha_p \geq\ 0$ *such that*

$${T_0} - \sum\limits_{i = 1}^p {\alpha _i}{T_i} \le 0,$$

*then the following is true*

$${\phi _1}(\varsigma ) \le 0,\;{\phi _2}(\varsigma ) \le 0,\; \cdots ,\;{\phi _p}(\varsigma ) \le 0 \Rightarrow {\phi _0}(\varsigma ) \le 0.$$

**Lemma 2** *(Schur Complement Lemma) Given constant matrices S*_*1*_, *S*_*2*_ *and S*_*3*_ *where* ${S_1} = S_1^T$ *and* $0 < {S_2} = S_2^T$, *then* ${S_1} + S_3^TS_2^{ - 1}{S_3} < 0$ *if and only if*

$$\left[ {\matrix{ {{S_1}} & {S_3^T} \cr {{S_3}} & { - {S_2}} \cr } } \right] < 0\quad or\quad \left[ {\matrix{ { - {S_2}} & {{S_3}} \cr {S_3^T} & {{S_1}} \cr } } \right] < 0.$$

**Lemma 3** (Boyd et al., 1994) *Let A = A*^*T*^, *and M, N be real matrices of appropriate dimensions with LL*^*T*^*≤ I. Then, A + MLN + N*^*T*^*L*^*T*^*M*^*T*^*≤ 0, if and only if there exists a positive scalar κ > 0 such that A + κ MM*^*T*^ *+ κ*^*−1*^*N*^*T*^*N ≤ 0 or equivalently*

$$\left[ {\matrix{ A & {\kappa M} & {{N^T}} \cr {\kappa {M^T}} & { - \kappa I} & 0 \cr N & 0 & { - \kappa I} \cr } } \right] \le 0.$$

For the purpose of simplicity, set

$$\matrix{ {}{\delta (k) \triangleq  {{[\delta _1^T(k)\;\;\delta _2^T(k)\;\; \cdots \;\;\delta _N^T(k)]}^T},\quad \quad \quad \;\;{\hat {\bar x}(k)} \triangleq {\kern 1pt} {{[{\hat {\bar x}_1^T(k)}\;\;{\hat {\bar x}_2^T(k)}\;\; \cdots \;\;{\hat {\bar x}_N^T(k)}]}^T},} \cr {}{\tilde x(k) \triangleq {\kern 1pt} {{[\tilde x_1^T(k)\;\;\tilde x_2^T(k)\;\; \cdots \;\;\tilde x_N^T(k)]}^T},\quad \quad \quad \quad \vec x(k) \triangleq {\kern 1pt} {{[\underbrace{{{{\bar x}^T}(k)\;\;{{\bar x}^T}(k)\;\; \cdots \;\;{{\bar x}^T}(k)}}_{N}]}^T},} \cr {{\rm {\mathcal{D}}}(k) \triangleq {\kern 1pt} {\rm diag}\{ {{\bar D}_1}(k),\;\;{{\bar D}_2}(k),\;\; \cdots ,\;\;{{\bar D}_N}(k)\} ,\quad \;{\rm {\mathcal{E}}}(k) \triangleq {\kern 1pt} {\rm diag}\{ {E_1}(k),\;\;{E_2}(k),\;\; \cdots ,\;\;{E_N}(k)\} .}\cr {} & {}\cr }$$

Then, the estimation error system (11) can be rewritten as the following compact form

(14) $$\eqalign{ \tilde x(k + 1) = {\kern 1pt} ({I_N} \otimes \bar A(k) - {\rm {\mathcal{G}}}(k){\rm {\mathcal{D}}}(k))\tilde x(k) + ({I_N} \otimes \Delta\bar A(k))\vec x(k) + ({{\bf 1}_N} \otimes \bar C(k))v(k) \cr- {\rm {\mathcal{G}}}(k)\delta (k){\kern 1pt} - {\rm {\mathcal{G}}}(k){\rm {\mathcal{E}}}(k)({{\bf 1}_N} \otimes {I_{{n_w}}})w(k)}$$

where ${\rm {\mathcal{G}}}(k) = [{\bar G_{ij}}(k{)]_{N \times N}} \in {{\rm {\mathbb R}}^{{n_f}N \times pN}}$ with

$${\bar G_{ij}}(k) = \left\{ {\matrix{ {} \hfill {{a_{ij}}{G_{ij}}(k),} \hfill  {i = 1,2, \ldots ,N;\quad j \in {{\rm {\mathcal{N}}}_i}} \hfill \cr {} \hfill {0,} \hfill  {i = 1,2, \ldots ,N;\quad j\ \notin\ {{\rm {\mathcal{N}}}_i}.} \hfill \cr } } \right.$$

In the following theorem, a sufficient condition is presented to guarantee that the dynamics of the estimation error ${\tilde x_i}(k),\; i = 1, 2,{\ldots},N$ satisfies the constraint (13).

**Theorem 3.1** *Consider the time-varying system* (1) *and* (2), *the fault* (6) *and the estimator* (9). *Given the sequence of constraint matrices P(k) > 0, suppose that there exist real-valued matrices* ${\rm {\mathcal{G}}}(k),$ *positive scalars* $\alpha_{1,i}(k),\; \alpha_{2,i}(k)\; (i = 1,2,{\ldots},N),\; \alpha_{3}(k),\; \alpha_{4}(k)$ *and κ satisfying the following recursive linear matrix inequalities*

(15) $$\left[ {\matrix{ { - \Lambda (k)}  & {{\Xi ^T}(k){\rm {\mathcal{H}}}_{{n_f},i}^T} & 0 & {{{\bar {\rm {\!\!\mathcal{N}}}}^T}(k)} \cr {{{\rm {\mathcal{H}}}_{{n_f},i}}\Xi (k)} & { - P(k + 1)} & {\kappa {{\rm {\mathcal{H}}}_{{n_f},i}}{\rm {\mathcal{M}}}(k)} & 0 \cr 0 & {\kappa {{\rm {\mathcal{M}}}^T}(k){\rm {\mathcal{H}}}_{{n_f},i}^T} & { - \kappa I} & 0 \cr {\bar{\rm {\!\!\mathcal{N}}}(k)} & 0 & 0 & { - \kappa I} \cr } } \right] \le 0,\;\;(i = 1,2, \ldots ,N)$$

*where*

$$\Lambda (k) = \;{\rm diag}\Big\{ \bar \alpha (k),{\kern 1pt} \sum\limits_{i = 1}^N {\alpha _{1,i}}(k){\rm {\mathcal{H}}}_{r,i}^T{{\rm {\mathcal{H}}}_{r,i}},{\kern 1pt} \sum\limits_{i = 1}^N {\alpha _{2,i}}(k){\bar \varepsilon ^{\; - 1}}{\rm {\mathcal{H}}}_{p,i}^T{{\rm {\mathcal{H}}}_{p,i}},{\kern 1pt} {\alpha _3}(k){S^{ - 1}}(k),{\kern 1pt} {\alpha _4}(k){T^{ - 1}}(k)\Big\} ,$$

$$\bar \alpha (k) = \;1 - \sum\limits_{i = 1}^N ({\alpha _{1,i}}(k) + {\alpha _{2,i}}(k)) - {\alpha _3}(k) - {\alpha _4}(k),\quad {{\rm {\mathcal{H}}}_{\iota ,i}} = \;({\bf 1}_N^T \otimes {I_\iota }){\Upsilon _{\iota ,i}} \in {{\rm {\mathbb R}}^{\iota \times N\iota }},$$

$${\Upsilon _{\iota ,i}} = \;{\rm diag}\{ \underbrace{{{0_\iota },\; \cdots {{,0}_\iota }}}_{{i - 1}},\;{I_\iota },\;\underbrace{{{0_\iota },\; \cdots {{,0}_\iota }}}_{{N - i}}\} ,\iota = \{ {n_f},r,p\} ,$$

$$\Xi (k) = \;\left[ {0,\;{\Xi _1}(k),\; - {\rm {\mathcal{G}}}(k),\;{{\bf 1}_N} \otimes \bar C(k),\;{\Xi _2}(k)} \right],$$

$${\Xi _1}(k) = \;{I_N} \otimes (\bar A(k)R(k)) - {\rm {\mathcal{G}}}(k){\rm {\mathcal{D}}}(k)({I_N} \otimes R(k)),$$

$${\Xi _2}(k) = \; - {\rm {\mathcal{G}}}(k){\rm {\mathcal{E}}}(k)({{\bf 1}_N} \otimes {I_{{n_w}}}),\quad \bar {\rm {\!\!\mathcal{N}}}(k) = \;{\rm {\mathcal{N}}}(k)[{\hat {\bar x}(k)},{\kern 1pt} {\rm {\mathcal{R}}}(k),{\kern 1pt} 0,{\kern 1pt} 0,{\kern 1pt} 0],$$

$${\rm {\mathcal{M}}}(k) = \;{I_N} \otimes \bar M(k),\;{\rm {\mathcal{N}}}(k) = \;{I_N} \otimes \bar N(k),\;{\rm {\mathcal{R}}}(k) = \;{I_N} \otimes R(k)$$

*with* $R(k) \in {{\rm {\mathbb R}}^{{n_f} \times r}}$ *being a factorization of P*(*k*) (*i.e*., *P*(*k*) = *R*(*k*)*R*^*T*^(*k*)). *Then, the dynamics of the system* (14) *satisfies the constraint* (13).

*Proof*. We will prove this theorem by mathematical induction. First, it can be seen from Assumption 2.3 that

(16) $${\Psi _i}(0) = \tilde x_i^T(0){P^{ - 1}}(0){\tilde x_i}(0) = {\bar x^T}(0){P^{ - 1}}(0)\bar x(0) \le 1,\quad i = 1,2, \ldots ,N.$$

Assuming that *ψ*_*i*_(*k*) ≤ 1 is true, we need to show that *ψ*_*i*_(*k* + 1) ≤ 1 holds.

Since *ψ*_*i*_(*k*) ≤ 1 and *P*(*k*) = *R*(*k*)*R*^*T*^(*k*), it follows from Ghaoui & Calafiore (2001) that there exists ${s_i}(k) \in {{\rm {\mathbb R}}^r}$ $(i = 1,2,{\ldots},N)$ with *s*_*i*_(*k*) ≤ 1 satisfying

(17) $${\tilde x_i}(k) = R(k){s_i}(k).$$

Setting $s(k) \triangleq {[s_1^T(k)\;\;s_2^T(k)\;\; \cdots \;\;s_N^T(k)]^T},$ we have

(18) $$\tilde x(k) = ({I_N} \otimes R(k))s(k)$$

and

(19) $$\vec x(k) = {\hat {\bar x}(k)} + ({I_N} \otimes R(k))s(k).$$

Therefore, the estimation error system (14) can be written as

(20) $$\eqalign{ \tilde x(k + 1) = {\kern 1pt} ({I_N} \otimes ((\bar A(k) + \Delta\bar A(k))R(k)) - {\rm {\mathcal{G}}}(k){\rm {\mathcal{D}}}(k)({I_N} \otimes R(k)))s(k) \cr+ ({I_N} \otimes \Delta\bar A(k)){\hat {\bar x}(k)} + ({{\bf 1}_N} \otimes \bar C(k))v(k) - {\rm {\mathcal{G}}}(k)\delta (k) \cr- {\rm {\mathcal{G}}}(k){\rm {\mathcal{E}}}(k)({{\bf 1}_N} \otimes {I_{{n_w}}})w(k).}$$

Considering $\Xi (k) = \left[ {0,\;{\Xi _1}(k),\; - {\rm {\mathcal{G}}}(k),\;{{\bf 1}_N} \otimes \bar C(k),\;{\Xi _2}(k)} \right]$ with $\Xi_1(k), \Xi_2(k)$ defined in (15), and denoting

$$\xi (k) \triangleq {\left[ {1,\;{s^T}(k),\;{\delta ^T}(k),\;{v^T}(k),\;{w^T}(k)} \right]^T},\quad \Delta\Xi (k) \triangleq \left[ {\Delta{\Xi _1}(k),\;\Delta{\Xi _2}(k),\;0,\;0,\;0} \right]$$

where $\Delta{\Xi _1}(k) = ({I_N} \otimes \Delta\bar A(k)){\hat {\bar x}(k)}$ and $\Delta{\Xi _2}(k) = ({I_N} \otimes \Delta\bar A(k){R(k))}$, then (20) can be rewritten as

(21) $$\tilde x(k + 1) = (\Xi (k) + \Delta\Xi (k))\xi (k).$$

According to the definition of ${\rm {\mathcal{M}}}(k)$ and $\bar {\rm {\!\!\mathcal{N}}}(k)$ in (15), it is easy to verify that

(22) $$\Delta\Xi (k) = {\rm {\mathcal{M}}}(k){\rm {\mathcal{L}}}(k)\ \bar{\rm {\!\!\mathcal{N}}}(k)$$

where ${\rm {\mathcal{L}}}(k) = {I_N} \otimes \bar L(k)$ which satisfies that ${{\rm {\mathcal{L}}}^T}(k){\rm {\mathcal{L}}}(k) \le I.$

It follows from (21) that

(23) $${\tilde x_i}(k + 1) = {{\rm {\mathcal{H}}}_{{n_f},i}}(\Xi (k) + \Delta\Xi (k))\xi (k).$$

Then, one obtains

(24) $$\eqalign{ \tilde x_i^T(k + 1){P^{ - 1}}(k + 1){\tilde x_i}(k + 1) - 1 = \;{\xi ^T}(k)[(\Xi (k) + \Delta\Xi (k{))^T} \cr {\rm {\mathcal{H}}}_{{n_f},i}^T{P^{ - 1}}(k + 1){{\rm {\mathcal{H}}}_{{n_f},i}}(\Xi (k) + \Delta\Xi (k)) - {\rm diag}\{ 1,{\kern 1pt} 0,{\kern 1pt} 0,{\kern 1pt} 0,{\kern 1pt} 0\} ]\xi (k).}$$

According to Assumption 2.1, (12) and (17), we have

(25) $$\left\{ {\matrix{ {{\rm \parallel }{s_i}(k){{\rm \parallel }^2} \le 1,} \hfill \cr {} {{{\bar \varepsilon }^{\; - 1}}{\rm \parallel }{\delta _i}(k){{\rm \parallel }^2} \le 1,} \hfill \cr {} {{v^T}(k){S^{ - 1}}(k)v(k) \le 1,} \hfill \cr {} {{w^T}(k){T^{ - 1}}(k)w(k) \le 1,} \hfill \cr } } \right.$$

which can be rearranged in terms of *ξ*(*k*) as follows:

(26) $$\left\{ {\matrix{ {} {{\xi ^T}(k){\rm diag}\{ - 1,{\kern 1pt} {\rm {\mathcal{H}}}_{r,i}^T{{\rm {\mathcal{H}}}_{r,i}},{\kern 1pt} 0,{\kern 1pt} 0,{\kern 1pt} 0\} \xi (k) \le 0,} \hfill \cr {} {{\xi ^T}(k){\rm diag}\{ - 1,{\kern 1pt} 0,{\kern 1pt} {{\bar \varepsilon }^{\; - 1}}{\rm {\mathcal{H}}}_{p,i}^T{{\rm {\mathcal{H}}}_{p,i}},{\kern 1pt} 0,{\kern 1pt} 0\} \xi (k) \le 0,} \hfill \cr {} {{\xi ^T}(k){\rm diag}\{ - 1,{\kern 1pt} 0,{\kern 1pt} 0,{\kern 1pt} {S^{ - 1}}(k),{\kern 1pt} 0\} \xi (k) \le 0,} \hfill \cr {} {{\xi ^T}(k){\rm diag}\{ - 1,{\kern 1pt} 0,{\kern 1pt} 0,{\kern 1pt} 0,{\kern 1pt} {T^{ - 1}}(k)\} \xi (k) \le 0.} \hfill \cr } } \right.$$

It can be known from Lemma 1 that, if there exist non-negative scalars *α*_1,*i*_(*k*), *α*_2,*i*_(*k*) *i* = (1,2,…,*N*), *α*_3_(*k*) and *α*_4_(*k*) such that

$${(\Xi (k) + \Delta\Xi (k))^T}{\rm {\mathcal{H}}}_{{n_f},i}^T{P^{ - 1}}(k + 1){{\rm {\mathcal{H}}}_{{n_f},i}}(\Xi (k) + \Delta\Xi (k)) - {\rm diag}\{ 1,{\kern 1pt} 0,{\kern 1pt} 0,{\kern 1pt} 0,{\kern 1pt} 0\}$$

$$- \sum\limits_{i = 1}^N {\alpha _{1,i}}(k){\rm diag}\{ - 1,{\kern 1pt} {\rm {\mathcal{H}}}_{r,i}^T{{\rm {\mathcal{H}}}_{r,i}},{\kern 1pt} 0,{\kern 1pt} 0,{\kern 1pt} 0\} - \sum\limits_{i = 1}^N {\alpha _{2,i}}(k){\rm diag}\{ - 1,{\kern 1pt} 0,{\kern 1pt} {\bar \varepsilon ^{\; - 1}}{\rm {\mathcal{H}}}_{p,i}^T{{\rm {\mathcal{H}}}_{p,i}},{\kern 1pt} 0,{\kern 1pt} 0\}$$

(27) $$- {\alpha _3}(k){\rm diag}\{ - 1,{\kern 1pt} 0,{\kern 1pt} 0,{\kern 1pt} {S^{ - 1}}(k),{\kern 1pt} 0\} - {\alpha _4}(k){\rm diag}\{ - 1,{\kern 1pt} 0,{\kern 1pt} 0,{\kern 1pt} 0,{\kern 1pt} {T^{ - 1}}(k)\} \le 0$$

then the following inequality

(28) $${(\Xi (k) + \Delta\Xi (k))^T}{\rm {\mathcal{H}}}_{{n_f},i}^T{P^{ - 1}}(k + 1){{\rm {\mathcal{H}}}_{{n_f},i}}(\Xi (k) + \Delta\Xi (k)) - {\rm diag}\{ 1,{\kern 1pt} 0,{\kern 1pt} 0,{\kern 1pt} 0,{\kern 1pt} 0\} \le 0$$

holds resulting from (26). Moreover, the inequality (27) can be rewritten as

(29) $${(\Xi (k) + \Delta\Xi (k))^T}{\rm {\mathcal{H}}}_{{n_f},i}^T{P^{ - 1}}(k + 1){{\rm {\mathcal{H}}}_{{n_f},i}}(\Xi (k) + \Delta\Xi (k)) - \Lambda (k) \le 0$$

where *λ*(*k*) is defined in (15).

By using Lemma 2, (29) is equivalent to

(30) $$\left[ {\matrix{ { - \Lambda (k)} & {{{(\Xi (k) + \Delta\Xi (k))}^T}{\rm {\mathcal{H}}}_{{n_f},i}^T} \cr {{{\rm {\mathcal{H}}}_{{n_f},i}}(\Xi (k) + \Delta\Xi (k))} & { - P(k + 1)} \cr } } \right] \le 0$$

which follows from (22) that

(31) $$\eqalign{ \left[ {\matrix{ { - \Lambda (k)} & {{\Xi ^T}(k){\rm {\mathcal{H}}}_{{n_f},i}^T} \cr {{{\rm {\mathcal{H}}}_{{n_f},i}}\Xi (k)} & { - P(k + 1)} \cr } } \right] + \left[ {\matrix{ 0 \cr {{{\rm {\mathcal{H}}}_{{n_f},i}}{\rm {\mathcal{M}}}(k)} \cr } } \right]{\rm {\mathcal{L}}}(k)\left[ {\matrix{ {\bar {\rm {\!\!\mathcal{N}}}(k)}  0 \cr } } \right] \cr + \left[ {\matrix{ \ {{\bar {{\rm {\!\!\mathcal{N}}}}^T}(k)} \cr 0 \cr } } \right]{{\rm {\mathcal{L}}}^T}(k)\left[ {\matrix{ 0  {{{\rm {\mathcal{M}}}^T}(k){\rm {\mathcal{H}}}_{{n_f},i}^T} \cr } } \right] \le 0.}$$

According to Lemma 3, the inequality (31) holds if and only if there exists a positive scalar *κ* > 0 such that

$$\left[ {\matrix{ { - \Lambda (k)} & {{\Xi ^T}(k){\rm {\mathcal{H}}}_{{n_f},i}^T} & 0 & {{{\bar {\rm {\!\!\mathcal{N}}}}^T}(k)} \cr {{{\rm {\mathcal{H}}}_{{n_f},i}}\Xi (k)} & { - P(k + 1)} & {\kappa {{\rm {\mathcal{H}}}_{{n_f},i}}{\rm {\mathcal{M}}}(k)} & 0 \cr 0 & {\kappa {{\rm {\mathcal{M}}}^T}(k){\rm {\mathcal{H}}}_{{n_f},i}^T} & { - \kappa I} & 0 \cr {\bar {\rm {\!\!\mathcal{N}}}(k)} & 0 & 0 & { - \kappa I} \cr } } \right] \le 0.$$

Hence, it is obtained that the inequality (28) holds if RLMI (15) is satisfied. Considering (24), we have

$${\Psi _i}(k + 1) = \tilde x_i^T(k + 1){P^{ - 1}}(k + 1){\tilde x_i}(k + 1) \le 1$$

and the induction is accomplished. Therefore, the dynamics of the system (14) satisfies the constraint (13) with the estimator gain ${\rm {\mathcal{G}}}(k),$ which completes the proof.

It can be observed that the constraint (13) is equivalent to the quadratic error-bounded constraint $\tilde x_i^T(k){\tilde x_i}(k) \le P(k),$ *i* = 1, 2, …, *N*. In order to minimize *P*(*k*), the following corollary is proposed which may help the design the locally optimal estimator gains.

**Corollary 1.** *Consider the time-varying system* (1) *and* (2), *the fault* (6) *and the estimator* (9). *The ellipsoid constraint P(k) is minimized (in the sense of matrix trace) if there exist real-valued matrices* ${\rm {\mathcal{G}}}(k),$ *positive scalars α*_*1,i*_*(k), α*_*2,i*_*(k) (i = 1,2,…,N), α*_*3*_*(k), α*_*4*_*(k) and κ solving the following optimization problem*

(32) $$\mathop {\min }\limits_{P(k + 1),{\kern 1pt} {\rm {\mathcal{G}}}(k),{\kern 1pt} {\alpha _{1,i}}(k),{\kern 1pt} {\alpha _{2,i}}(k),{\kern 1pt} {\alpha _3}(k),{\kern 1pt} {\alpha _4}(k),{\kern 1pt} \kappa } {\rm tr}\{ P(k + 1)\}$$

*subject to* (15).

**Remark 3.** *Corollary 1 gives a method to design an ellipsoid of the smallest size to constrain state estimates of all the sensor nodes by optimizing the trace of P(k + 1) at each time step. We can see that the inequality* (15) *is linear to the variables P(k + 1)*, ${\rm {\mathcal{G}}}(k),$ *α*_*1,i*_*(k), α*_*2,i*_*(k) α*_*3*_*(k), α*_*4*_*(k) and κ. Hence, the optimization problem* (32) *subject to* (15) *can be solved by the existing semi-definite programming *via* interior-point method* Nesterov & Nemirovski (1994). *The trace of P(k + 1) is optimized at each time step in an effort to find the minimal ellipsoid*.

According to Theorem 3.1 and Corollary 1, the Set-membership Fault Estimator Design (SFED) algorithm can be summarized as follows.

**Remark 4.** *It is worth noting that the condition* (15) *in Theorem 3.1 contains all the information of the plant dynamics including the bounded of unknown noises, time-varying systems parameters, quantizing level as well as communication topology. According to Algorithm SFED, the estimator parameters G*_*ij*_*(k) can be computed by solving a set of RLMIs, and such a recursive process is suitable for online application*.

From the global point of view, researchers are more interested in the performance in terms of the average of state estimation errors from all the sensor nodes rather than the individual ones Nesterov & Nemirovski (1994). Next, we will consider the fault estimation problem with constraints imposed on the average estimation error which is defined as follows

(33) $$\rho (k) = \sum\limits_{i = 1}^N {\sigma _i}{ (\bar x(k)} - { \hat{\bar x_i}}(k)) = \left( {{\bf 1}_N^T\Sigma \otimes {I_{{n_f}}}} \right)\tilde x(k)$$

where *$\Sigma$* = diag{*σ*_1_,*σ*_2_,⋯,*σ*_*N*_} with *σ*_*i*_ being the weighting parameters which denotes the priorities to the corresponding node.

Suppose *ρ*^*T*^(0)*Q* ^−1^(0)*ρ*(0) ≤ 1, where *Q*(0) is a positive definite matrix. Given a sequence of constraint matrices *Q*(*k*), we need to design the estimator (9) such that the following inequality holds

(34) $${\rho ^T}(k){Q^{ - 1}}(k)\rho (k) \le 1,\quad k \in {\rm {\mathbb N}}.$$

**Theorem 3.2** *Consider the time-varying system* (1) and (2), *the fault* (6) *and the estimator* (9). *Given the sequence of constraint matrices Q(k) > 0, the requirement* (34) *is realized if there exist real-valued matrices* ${\rm {\mathcal{G}}}(k),$ *positive scalars α*_*1,i*_*(k), α*_*2,i*_*(k) (i = 1,2,…,N), α*_*3*_*(k), α*_*4*_*(k) and κ satisfying the following RLMIs:*

(35) $$\left[ {\matrix{ { - \Lambda (k)} &  {{\Xi ^T}(k){{\left( {{\bf 1}_N^T\Sigma \otimes {I_{{n_f}}}} \right)}^T}} & 0 & {{{\bar {\rm {\!\!\mathcal{N}}}}^T}(k)} \cr {\left( {{\bf 1}_N^T\Sigma \otimes {I_{{n_f}}}} \right)\Xi (k)} & { - Q(k + 1)} & {\kappa {{\rm {\mathcal{H}}}_{{n_f},i}}{\rm {\mathcal{M}}}(k)} & 0 \cr 0 & {\kappa {{\rm {\mathcal{M}}}^T}(k){\rm {\mathcal{H}}}_{{n_f},i}^T} & { - \kappa I} & 0 \cr {{\bar \rm {\!\!\mathcal{N}}}(k)} & 0 & 0 & { - \kappa I} \cr } } \right] \le 0\;\;(i = 1,2, \ldots ,N)$$

where Λ(*k*), ξ(*k*), ${\rm {\mathcal{M}}}(k),$ $\bar {\rm {\!\!\mathcal{N}}}(k)$ and ${{\rm {\mathcal{H}}}_{{n_f},i}}$ are the same as defined in (15).

*Proof*. Considering (21) and (33), it is easy to verify that

$${\rho ^T}(k){Q^{ - 1}}(k)\rho (k) \le 1,$$

$$\Leftrightarrow {\tilde x^T}(k){\left( {{\bf 1}_N^T\Sigma \otimes {I_{{n_f}}}} \right)^T}{Q^{ - 1}}(k)\left( {{\bf 1}_N^T\Sigma \otimes {I_{{n_f}}}} \right)\tilde x(k) \le 1$$

$$\Leftrightarrow {\xi ^T}(k)(\Xi (k) + \Delta\Xi (k{))^T}{\left( {{\bf 1}_N^T\Sigma \otimes {I_{{n_f}}}} \right)^T}{Q^{ - 1}}(k + 1)\left( {{\bf 1}_N^T\Sigma \otimes {I_{{n_f}}}} \right)(\Xi (k) + \Delta\Xi (k))\xi (k) \le 1$$

$$\Leftrightarrow {\xi ^T}(k)(\Xi (k) + \Delta\Xi (k{))^T}{\left( {{\bf 1}_N^T\Sigma \otimes {I_{{n_f}}}} \right)^T}{Q^{ - 1}}(k + 1)\left( {{\bf 1}_N^T\Sigma \otimes {I_{{n_f}}}} \right)(\Xi (k) + \Delta\Xi (k))\xi (k)$$

(36) $$- {\xi ^T}(k){\rm diag}\{ 1,{\kern 1pt} 0,{\kern 1pt} 0,{\kern 1pt} 0,{\kern 1pt} 0\} \xi (k) \le 0.$$

By the similar procedure as in the proof of Theorem 3.1, it can be concluded that the objective (34) is implied by the condition (35). The proof is complete.

Consider the following time-varying system

(37) $$\left\{ {\matrix{ {x(k + 1) = (A(k) + \Delta A(k))x(k) + B(k)f(k) + C(k)v(k)} \cr {}{y(k) = q(D(k)x(k)) + E(k)w(k),} \cr } } \right.$$

and the estimator as follows:

(38) $${\hat {\bar x}(k + 1)} = {\kern 1pt} \bar A(k){\hat {\bar x}(k)} + G(k)(y(k) - \bar D(k){\hat {\bar x}(k)}),\quad {\hat {\bar x}(0)} = 0$$

where $G(k) \in {{\rm {\mathbb R}}^{{n_f} \times p}}$ is the estimation parameter to be determined.

**Corollary 2.** *Consider the time-varying system* (37), *the fault* (6) *and the estimator* (38). *Given the sequence of constraint matrices P(k) > 0, suppose that there exist real-valued matrices G(k), positive scalars α*_*1*_*(k), α*_*2*_*(k), α*_*3*_*(k), α*_*4*_*(k) and κ satisfying the following recursive linear matrix inequality*

(39) $$\left[ {\matrix{ { - \hat \Lambda (k)} & {{{\hat \Xi }^T}(k)} & 0 & {{{\hat N}^T}(k)} \cr {\Xi (k)} & { - P(k + 1)} & {\kappa \bar M(k)} & 0 \cr 0 & {\kappa {{\bar M}^T}(k)} & { - \kappa I} & 0 \cr {\hat N(k)} & 0 & 0 & { - \kappa I} \cr } } \right] \le 0$$

*where*

$$\hat \Lambda (k) = \;{\rm diag}\Big\{ \hat \alpha (k),{\kern 1pt} {\alpha _1}(k),{\kern 1pt} {\alpha _2}(k){\bar \varepsilon ^{\; - 1}},{\kern 1pt} {\alpha _3}(k){S^{ - 1}}(k),{\kern 1pt} {\alpha _4}(k){T^{ - 1}}(k)\Big\} ,$$

$$\hat \alpha (k) = \;1 - {\alpha _1}(k) - {\alpha _2}(k) - {\alpha _3}(k) - {\alpha _4}(k),\quad \hat N(k) = \;\bar N(k)[{\hat {\bar x}(k)},{\kern 1pt} R(k),{\kern 1pt} 0,{\kern 1pt} 0,{\kern 1pt} 0],$$

$$\hat \Xi (k) = \;[0,\;(\bar A(k) - G(k)\bar D(k))R(k),\; - {\rm {\mathcal{G}}}(k),\;\bar C(k),\; - G(k)E(k)],$$

with $R(k) \in {{\rm {\mathbb R}}^{{n_f} \times r}}$ being a factorization of *P*(*k*) (*i.e*., *P*(*k*) = *R*(*k*)*R*^*T*^(*k*)). Then, the dynamics of the system (37) satisfies the constraint ${(\bar x(k) - {\hat {\bar x}(k)})^T}{P^{ - 1}}(k)(\bar x(k) - {\hat {\bar x}(k)}) \le 1$.

**Remark 5.** *To our best knowledge, the results in this paper are still new even for time-varying system* (37) *with only one sensor node. Similar to Corollary 1, the minimized P(k) (in the sense of matrix trace) is guaranteed if there exist real-valued matrices G(k), positive scalars α*_*1*_*(k), α*_*2*_*(k), α*_*3*_*(k), α*_*4*_*(k) and κ solving the following optimization problem*

(40) $$\mathop {\min }\limits_{P(k + 1),{\kern 1pt} {\rm {\mathcal{G}}}(k),{\kern 1pt} {\alpha _1}(k),{\kern 1pt} {\alpha _2}(k),{\kern 1pt} {\alpha _3}(k),{\kern 1pt} {\alpha _4}(k),{\kern 1pt} \kappa } {\rm tr}\{ P(k + 1)\}$$

subject to RLMI (39).

## Numerical Example

In this section, two numerical examples are given to illustrate the proposed set-membership fault estimation methods.

*Example 1:* Consider the time-varying system (37) with the following parameters:

$$A(k) = \left[ {\matrix{ {0.9 + 0.2\cos (0.6k)} & {0.02 + 0.01\cos (0.2k)} \cr {0.05} & {0.4 + 0.5\sin (0.2k)} \cr } } \right],\quad B(k) = \left[ {\matrix{ 0 \cr 1 \cr } } \right],$$

$$C(k) = \left[ {\matrix{ {0.5 - 0.02\sin (0.3k)} \cr {0.1 + 0.05\cos (0.5k)} \cr } } \right],\quad D(k) = [0.2 + 0.1\cos (k)\;\; - 0.3],\quad E(k) = - 0.2.$$

The matrix *F*(*k*) in (6) is assumed to be

(41) $$F(k) = \left\{ {\matrix{ I & \hfill  {0 \le k \le 29} \hfill \cr {0.5I} & \hfill  {k = 30} \hfill \cr I \hfill & {31 \le k \le 59} \hfill \cr { - 2I} & \hfill  {k = 60} \hfill \cr I  & \hfill  {{\rm else}} \hfill \cr } } \right.$$

and the fault to be estimated is *f*(*k*) = 1. Moreover, the parameter uncertainties are supposed to be

$$\Delta A(k)=M_A(k) L_A(k) N_A(k),\; \Delta F(k)=M_F(k) L_F(k) N_F(k)$$

where *M*_*A*_(*k*) = [0.02 0.05]^*T*^, *L*_*A*_(*k*) = sin(*k*), *N*_*A*_(*k*) = [0.03 0.05] and *M*_*F*_(*k*) = 0.02, *L*_*F*_(*k*) = cos(*k*), *N*_*F*_(*k*) = 0.03. The bounded noises are assumed to be *v*(*k*) = 0.6cos(*k*) and *w*(*k*) = 0.6sin(*k*), which imply that *S*(*k*) = *T*(*k*) = 0.36*I*. The quantizing level is selected as *ε* = 0.1. Set $x(k) = {\left[ {{x^{(1)}}(k)\;\;{x^{(2)}}(k)} \right]^T}$ with *x*^(*s*)^(*k*) (*s* = 1,2) representing the *s*th entry of the vector *x*(*k*), and the initial conditions of *x*(*k*) and *P*(*k*) are chosen as follows: *x*(0) = [4 3]^*T*^, *P*(0) = diag{16,9,1}.

**Figure 1:**
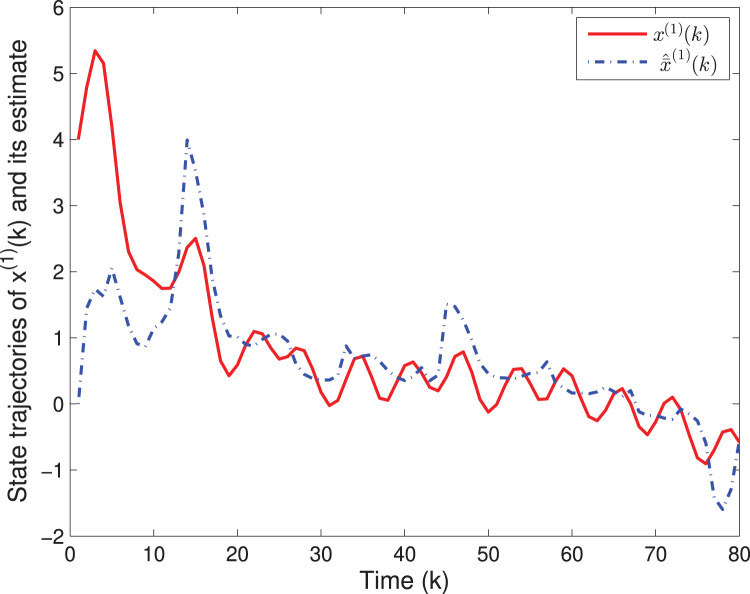
The state trajectories of *x*^(1)^(*k*) and *^x*^(1)^(*k*).

**Figure 2:**
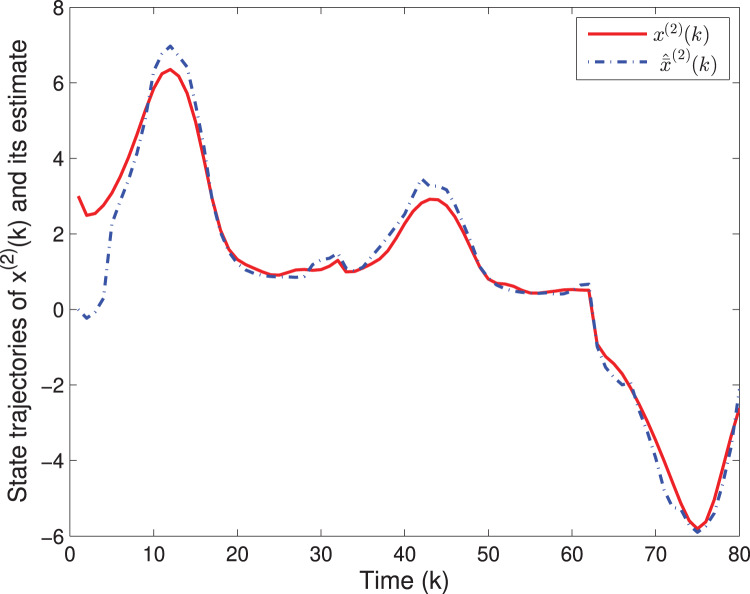
The state trajectories of *x*^(2)^(*k*) and *^x*^(2)^(*k*).

**Figure 3:**
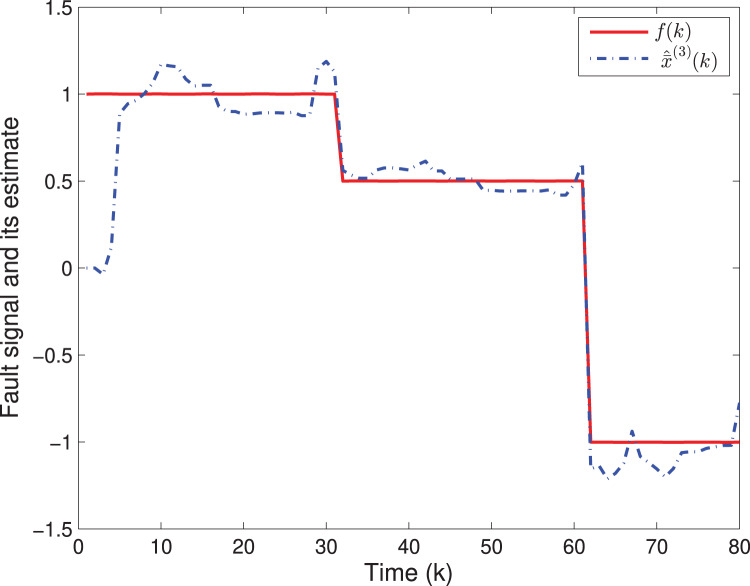
The fault signal *f*(*k*) and its estimate.

By means of Matlab software (YALMIP 3.0), the solution to the optimization problem (40) subject to (39) is derived, and the values of *G*(*k*) and *P*(*k*) are listed in Table 1. The simulation results are shown in Figs. 1–3. Figures 1 and 2 plot the state responses and the estimates of *x*^(1)^(*k*) and *x*^(2)^(*k*), respectively. Figure 3 depicts the fault signal *f*(*k*) and its estimate.

**Table 1:** The values of *G(k)* and *P(k)*.

*k*	*G*(*k*)	*P*(*k*)
0	$\left[ {\matrix{ {2.0654} \cr { - 0.3338} \cr 0 \cr } } \right]$	$\left[ {\matrix{ {16} & 0 & 0 \cr 0 & 9 & 0 \cr 0 & 0 & 1 \cr } } \right]$
1	$\left[ {\matrix{ {3.3071} \cr { - 0.3727} \cr { - 0.6047} \cr } } \right]$	$\left[ {\matrix{ {11.5622} & {3.6434} & { - 0.0001} \cr {3.6434} & {2.7857} & {1.2037} \cr { - 0.0001} & {1.2037} & {1.2097} \cr } } \right]$
2	$\left[ {\matrix{ {0.1484} \cr { - 0.7035} \cr { - 0.3917} \cr } } \right]$	$\left[ {\matrix{ {9.9897} & {4.7040} & {1.5882} \cr {4.7040} & {4.3026} & {2.1618} \cr {1.5882} & {2.1618} & {1.2850} \cr } } \right]$
3	$\left[ {\matrix{ { - 1.1009} \cr { - 3.1427} \cr { - 1.2926} \cr } } \right]$	$\left[ {\matrix{ {12.0708} & {5.9234} & {1.8607} \cr {5.9234} & {6.0813} & {2.6417} \cr {1.8607} & {2.6417} & {1.2670} \cr } } \right]$
$\vdots$	$\vdots$	$\vdots$
77	$\left[ {\matrix{ {1.4602} \cr {0.2848} \cr { - 0.0434} \cr } } \right]$	$\left[ {\matrix{ {10.6616} & {6.8340} & {1.0365} \cr {6.8340} & {5.6863} & {1.0619} \cr {1.0365} & {1.0619} & {0.2883} \cr } } \right]$
78	$\left[ {\matrix{ 0 \cr { - 0.0094} \cr { - 0.0036} \cr } } \right]$	$\left[ {\matrix{ {9.8534} & {6.4236} & {1.1481} \cr {6.4236} & {5.5303} & {1.2147} \cr {1.1481} & {1.2147} & {0.3539} \cr } } \right]$
79	$\left[ {\matrix{ { - 1.3181} \cr { - 2.0287} \cr { - 0.7918} \cr } } \right]$	$\left[ {\matrix{ {7.8414} & {4.8462} & {1.0077} \cr {4.8462} & {4.4368} & {1.1973} \cr {1.0077} & {1.1973} & {0.4046} \cr } } \right]$
80	$\left[ {\matrix{ { - 0.0138} \cr { - 0.0301} \cr { - 0.0158} \cr } } \right]$	$\left[ {\matrix{ {5.6749} & {2.6426} & {0.5611} \cr {2.6426} & {2.1194} & {0.6915} \cr {0.5611} & {0.6915} & {0.2981} \cr } } \right]$

**Algorithm:** SFED:

*Step 1*.	Set *k* = 0 and set the maximum step *k*_*m*_. Select the initial values of $\bar{x}(0)$ and *P*(0) satisfying Assumption 3. Set ${ \hat{ \bar x}_i}(0) = 0$, *i* = 1, 2, …, *N*, and ${ \hat{\bar x}(0)} = [ {\hat{\bar x}_1^T(0)}\; \cdots \; {\hat{\bar x}_N^T{(0)]^T}}$.
*Step 2*.	Compute the matrix *R*(*k*) by factorizing *P*(*k*).
*Step 3*.	With the known *R*(*k*) and $\hat{\bar x}(k),$ solve the optimization problem (32) subject to (15). If the optimization problem is solvable, *P*(*k*) and ${\rm {\cal G}}(k)$ can be derived. Then *G*_*ij*_(*k*) is known and go to the next step. Otherwise, this algorithm is infeasible. Stop.
*Step 4*.	Compute ${\hat {\bar x}_i}(k)$ according to (9) with the derived *G*_*ij*_(*k*). Then ${\hat{\bar x}(k)} = [{\hat {\bar x}_1^T(k)}\; \cdots \;{\hat {\bar x}_N^T(k{)]^T}}$ is obtained.
*Step 5*.	If *k* < *k*_*m*_, set *k* = *k* + 1 and go to Step 2. Otherwise, stop.

**Figure 4:**
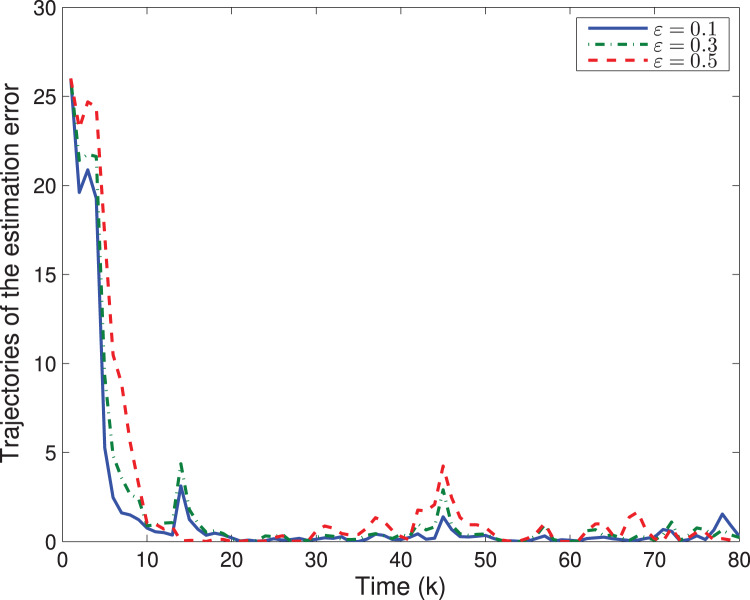
The trajectory of estimation error $\boldsymbol\|{\tilde {x}}(k)\|^2$ with different *ε*.

**Figure 5:**
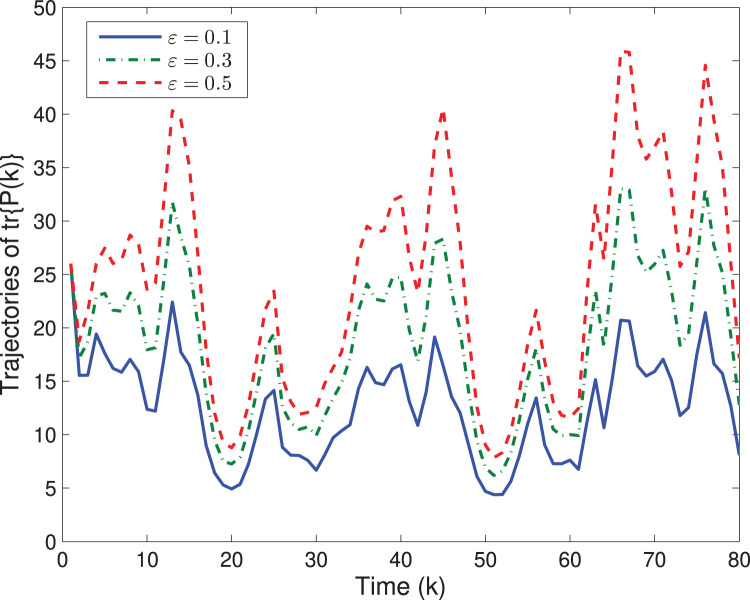
The trajectory of $\boldsymbol tr\{P(k)\}$ with different *ε*.

Next, let us examine how the quantizing level *ε* affects the system performances. By choosing different quantizing levels, the trajectories of the estimation error ${\rm \parallel }\tilde x(k){{\rm \parallel }^2}$ and tr{*P*(*k*)} are shown in Figs. 4, 5, respectively. It is easy to see that a larger *ε* would lead to a worse estimation error ${\rm \parallel }\tilde x(k){{\rm \parallel }^2}$ as well as a larger tr{*P*(*k*)}.

*Example 2:* Consider the time-varying system (1) with the following parameters:

$$A(k) = \left[ {\matrix{ {0.3 + 0.05\cos (0.6k)} & {0.02 + 0.01\cos (0.2k)} \cr {0.03} & {0.4 + 0.02\sin (0.2k)} \cr } } \right],$$

$$B(k) = \left[ {\matrix{ 0  {} \cr 1  {} \cr } } \right],\quad C(k) = \left[ {\matrix{ {0.3 + 0.05\sin (0.3k)}  {} \cr {0.1}  {} \cr } } \right].$$

**Figure 6:**
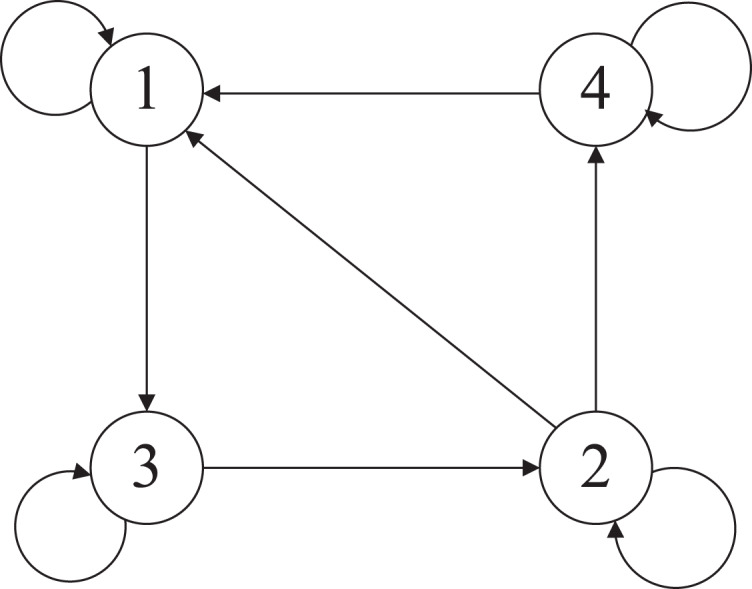
The directed communication graph.

Suppose that there are four sensing nodes with a directed communication graph ${\rm {\mathcal{O}}}$ shown in Fig. 6, and the adjacency elements associated with the edges of the graph are *a*_*ij*_ = 1. The parameters of the four sensor nodes modeled as (2) are as follows:

$${D_1}(k) = \;[0.2 + 0.1\cos (k)\quad - 0.3],\quad {E_1}(k) = \; - 0.02,$$

$${D_2}(k) = \;[0.3\quad 0.2 + 0.1\cos (k)],\quad \quad {E_2}(k) = \;0.03,$$

$${D_3}(k) = \;[0.2\quad 0.2 + 0.05\sin (k)],\quad \;\;\;{E_3}(k) = \;0.01 + 0.02\cos (k),$$

$${D_4}(k) = \;[0.15 + 0.1\sin (k)\quad 0.15],\quad \;{E_4}(k) = \; - 0.03.$$

**Figure 7:**
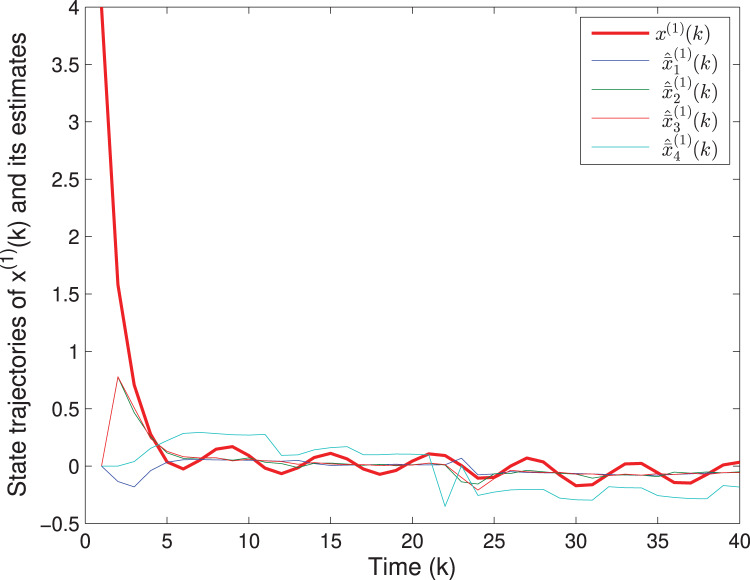
The state trajectories of $\boldsymbol x^{(\rm 1)}(k)$ and its estimates.

**Figure 8:**
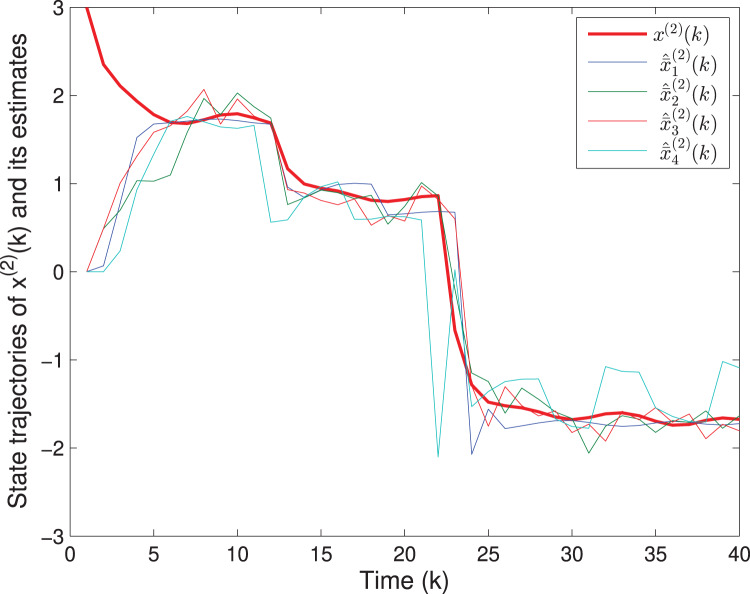
The state trajectories of $\boldsymbol x^{(\rm 2)}(k)$ and its estimates.

**Figure 9:**
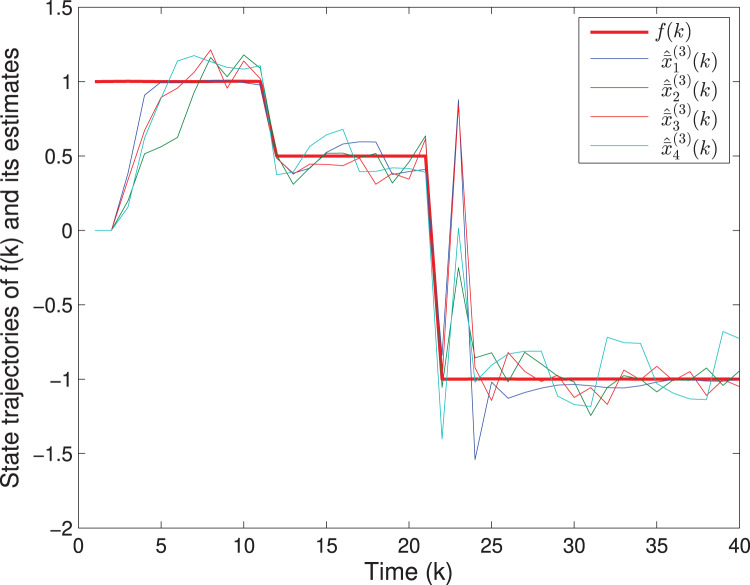
The fault signal $\boldsymbol f(k)$ and its estimates.

The matrix *F*(*k*) and the fault *f*(*k*) are the same as in Example 1. The uncertain parameters are chosen as *M*_*A*_(*k*) = [0.1 0.2]^*T*^, *L*_*A*_(*k*) = sin(*k*), *N*_*A*_(*k*) = [0.2 0.1] and *M*_*F*_(*k*) = 0.05, *L*_*F*_(*k*) = cos(*k*), *N*_*F*_(*k*) = 0.02. Assume that *v*(*k*) = 0.3cos(*k*) and *w*(*k*) = 0.2sin(*k*), which imply that *S*(*k*) = 0.09*I*, *T*(*k*) = 0.04*I*. The quantizing level is selected as *ε* = 0.1. The initial values are chosen as *x*(0) = [4 3]^*T*^ and *P*(0) = diag{16,9,1}. Based on Algorithm SFED and Matlab software (YALMIP 3.0), the optimization problem (32) subject to (15) is solved, and the results are shown in Figs. 7–9. Figures 7 and 8 plot the trajectories of the states *x*^(1)^(*k*), *x*^(2)^(*k*) and their estimates. Figure 9 depicts the fault signal and its estimates. The simulation results have confirmed that the fault estimation technology developed in this paper is indeed effective.

## Conclusion

In this article, the robust set-membership fault estimation problem has been investigated for time-varying systems with unknown-but-bounded noises and uniform quantization effects over sensor networks. By employing the mathematical induction method, a sufficient condition has been obtained under which the estimation error satisfies the prescribed constraint. An optimization problem has been proposed by minimizing the ellipsoid of the estimation error, and the time-varying parameters of the fault estimator have been derived by solving a set of RLMIs. Finally, two examples have been given to illustrate the effectiveness of the presented fault estimation approach.

## Supplemental Information

10.7717/peerj-cs.872/supp-1 Supplemental Information 1 The trajectory of estimation error ∥*x*(*k*)∥^2^ with different *ε*.

10.7717/peerj-cs.872/supp-2 Supplemental Information 2 The state responses and the estimates of *x*^(1)^(*k*) .

10.7717/peerj-cs.872/supp-3 Supplemental Information 3 The fault signal *f*(*k*) and its estimates.

10.7717/peerj-cs.872/supp-4 Supplemental Information 4 The state trajectories of *x*^(1)^(*k*) and its estimates.

10.7717/peerj-cs.872/supp-5 Supplemental Information 5 The state responses and the estimates of *x*^(2)^(*k*) .

10.7717/peerj-cs.872/supp-6 Supplemental Information 6 The directed communication graph.

10.7717/peerj-cs.872/supp-7 Supplemental Information 7 The state trajectories of *x*^(2)^(*k*) and its estimates.

10.7717/peerj-cs.872/supp-8 Supplemental Information 8 The fault signal *f*(*k*) and its estimate.

10.7717/peerj-cs.872/supp-9 Supplemental Information 9 The trajectory of tr{*P*(*k*)} with different *ε*.

10.7717/peerj-cs.872/supp-10 Supplemental Information 10 By means of Matlab software, the results of two numerical examples illustrated the effectiveness of the proposed fault estimator design scheme. Matlab software is required to view the code file.

## Additional Information and Declarations

### Data Availability

The following information was supplied regarding data availability: The raw data and code are available in the Supplemental Files.
